# Clinical Reflections

**Published:** 1883-02

**Authors:** Ad. Lippe

**Affiliations:** Philadelphia


### CLINICAL BUREAU.

### CLINICAL REFLECTIONS.

AD. LIPPE, M. D., PHILADELPHIA.

Mr. C., 16 years of age, coming to this city from Lexington, where he had lived through the summer, was taken sick in the middle of September with what seemed to be a malarial fever. One dose of Belladonna$^{cm}$ (Fincke) relieved him from the fever and violent headache. In a few days he was again able to leave the house. His former good appetite did not return, and on the 30th of September, 5 P. M., he had a chill, followed by fever and perspiration. On the 1st of October, 9 A. M., he had another severe chill, followed by fever and perspiration. I visited him first on October 1st, at 2 P. M.; he had no pains during these two irregular paroxysms, no thirst, no appetite, no sleep. I left one dose of China off.$^{cm}$ (Fincke), to be given him an hour after the perspiration had ceased, and this dose was given him at 8 P. M. At 9 P. M. he was found asleep and he slept soundly, without changing his position, until 9 A. M. On the 2d of October took some food and remained in bed that day. At 6 P. M. of the 2d and at 9 A. M. of the 3d of October he had a slight fever, but no chill nor any perspiration; no medicine. On the 4th and 5th of October he had still less fever at the same time as before. His appetite returned and he was able to go out on the 7th of October. There had not been the slightest return of it on the 17th of October.

*Comments:* The cases of intermittent fever similar to the symptoms caused by China off. are extremely rare, and as this remedy is so seldom indicated, it is of great interest to report one of these rare cases. In this particular case we find an irregularity seldom observed in the returns of the paroxysms—one day late in the day and the following day early in the day; the periodicity, therefore, gave no such a direct indication as we find under other remedies, nor were there present any of the frequent concomitant symptoms so often indicating a remedy; the only symptom was a negative one, viz.: "The entire absence of thirst during the paroxysm." The result, *i. e.*, a complete cure, showed the correctness of the choice of the remedy and also that it was correctly applied.

It is now ninety-two years (1790) since the immortal Hahnemann made his first proving of a drug, and that drug was the Cinchona bark, and he made it that he might answer the question asked by the honest and intelligent Dr. Cullen: "Under what circumstances would Cinchona bark cure intermittent fever?" Hahnemann, when he concluded to prove Cinchona bark on a healthy person to ascertain its sick-making properties, had then and there *a revelation;* then and there, by these means, he became conscious that the law of the Similars was the only law of cure. He made further experiments, and his consciousness became a conviction. Hahnemann published his *Materia Medica Pura*, and we find in the second edition of it, published in 1825, the pathogenesis of Cinchona off. with one of those prefaces characteristic of that diligent and honest healer. A very clever translation of this preface, which should be well studied by every true homœopath, is to be found in Dr. R. E. Dudgeon's translation of Hahnemann's *Materia Medica Pura* (page 408), 1880. That this preface, as well as other writings of the founder of our school, are utterly unknown to, and therefore are ignored by, a number of professing homœopaths, and, I am sorry to say, even by public teachers, even by teachers in *a* Hahnemann Medical College, is evidenced by what we find published in that professedly homœopathic journal, the *Hahnemannian Monthly*, the organ of the Hahnemann Club, of Philadelphia, and we regret very much to call the attention of the Hahnemann Club to that preface and to their unphilosophical, absurd utterances before the Philadelphia County Society, published in their own journal. They really glory in and boast of the vilest departures from the master's teaching and unblushingly expose their "testimonium paupertatis" to the world in the April number of their journal, *vide* page 205. With the exception of a feeble attempt to defend the master's teachings, his teachings were either unknown to, or were ignored by, these inventors of new auxiliary and supplementary principles, which till now, notwithstanding frequent polite petitions, they have never divulged. We now take it for granted that *one* of these principles is "the Hahnemann Club superior to Hahnemann." Proof, compare Hahnemann's preface to Cinchona and the Club's teachings.

